# Targeting Altered Energy Metabolism in Colorectal Cancer: Oncogenic Reprogramming, the Central Role of the TCA Cycle and Therapeutic Opportunities

**DOI:** 10.3390/cancers12071731

**Published:** 2020-06-29

**Authors:** Carina Neitzel, Philipp Demuth, Simon Wittmann, Jörg Fahrer

**Affiliations:** Division of Food Chemistry and Toxicology, Department of Chemistry, Technical University of Kaiserslautern, 67663 Kaiserslautern, Germany; cneitzel@rhrk.uni-kl.de (C.N.); pdemuth@rhrk.uni-kl.de (P.D.); wittmans@rhrk.uni-kl.de (S.W.)

**Keywords:** colorectal cancer, cancer metabolism, mitochondria, TCA cycle, oncogenic signaling, tumor microenvironment, targeted therapy, CPI-613

## Abstract

Colorectal cancer (CRC) is among the most frequent cancer entities worldwide. Multiple factors are causally associated with CRC development, such as genetic and epigenetic alterations, inflammatory bowel disease, lifestyle and dietary factors. During malignant transformation, the cellular energy metabolism is reprogrammed in order to promote cancer cell growth and proliferation. In this review, we first describe the main alterations of the energy metabolism found in CRC, revealing the critical impact of oncogenic signaling and driver mutations in key metabolic enzymes. Then, the central role of mitochondria and the tricarboxylic acid (TCA) cycle in this process is highlighted, also considering the metabolic crosstalk between tumor and stromal cells in the tumor microenvironment. The identified cancer-specific metabolic transformations provided new therapeutic targets for the development of small molecule inhibitors. Promising agents are in clinical trials and are directed against enzymes of the TCA cycle, including isocitrate dehydrogenase, pyruvate dehydrogenase kinase, pyruvate dehydrogenase complex (PDC) and α-ketoglutarate dehydrogenase (KGDH). Finally, we focus on the α-lipoic acid derivative CPI-613, an inhibitor of both PDC and KGDH, and delineate its anti-tumor effects for targeted therapy.

## 1. Introduction

Colorectal cancer (CRC) is one of the most prevalent malignant diseases with a global burden of about 1.8 million new cases every year [1]. Europe, North America and Australia show the highest age-standardized incidence rates, continuously rising in younger people over the last 25 years [2,3]. CRC is a multifactorial disease, which is closely linked to genetic predisposition, inflammatory bowel disease (IBD), smoking, alcohol consumption and dietary factors [1,4,5,6]. More than 50% of CRC patients are diagnosed with advanced disease (stage III and IV) [7,8,9]. CRC therapy is essentially based on surgery, radiotherapy and chemotherapy. Chemotherapy regimens include 5-Fluorouracil (5-FU) and folinic acid (leucovorin) as the gold standard, which are combined either with oxaliplatin (FOLFOX) or with irinotecan (FOLFIRI) [10]. In late stage metastatic disease, chemotherapy is performed together with targeted therapies, mainly aiming at the epidermal growth factor receptor (EGFR) and vascular endothelial growth factor (VEGF) [10]. Despite improved metastatic surgery and further treatment options, 5 year survival rates in late stage patients are still low [11], highlighting the need for novel therapeutic approaches. A field, which increasingly attracts attention, is the metabolic reprogramming of cancer cells during malignant transformation [12]. Mitochondria are central to this process, as will be detailed below. Our understanding of the specific metabolic alterations in cancer cells is continuously growing and has provided novel therapeutic targets for CRC treatment, which will be discussed in this review.

## 2. Mitochondrial Metabolism of Colorectal Cancer

**Figure 1:**
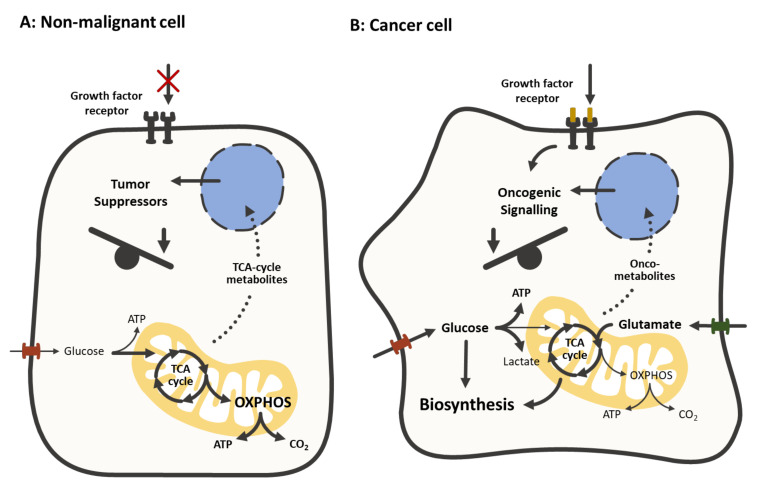
Comparison of the metabolism in non-malignant cells (**A**) and cancer cells (**B**) In most non-malignant cells, the absence of growth factor signaling, the limited supply of nutrients and the activity of tumor suppressors promotes oxidative phosphorylation (OXPHOS) in order to maximize the energetic yield. In cancer cells, growth factor signaling and oncogenes facilitate the transporter-mediated uptake of nutrients (e.g., glucose, glutamate) to supply a metabolic shift towards the biosynthesis of lipids, nucleotides and proteins. The TCA cycle serves as the main hub, connecting anabolic and catabolic processes by generating and providing intermediates. Metabolites and oncometabolites of the TCA cycle act as signaling molecules.

During the process of malignant transformation, a number of metabolic adaptions occur, either as a response to a changing tumor microenvironment (TME) and the corresponding bioenergetic demands or as a consequence of altered cellular growth signaling (Figure 1). Unarguably, the most extensively studied change of cancer bioenergetics is the so-called Warburg effect. This term was named after Otto Warburg, who first described the metabolic shift towards aerobic glycolysis as a primary energy source in cancer cells almost 100 years ago [13]. In contrast to untransformed cells, most cancer cells do not utilize oxidative phosphorylation (OXPHOS) to supply energy for cellular processes. Instead, they rely on glycolysis accompanied by the production of lactate even in the presence of oxygen [14].

In the meantime, several studies have underlined the importance of metabolic adaptions beyond the Warburg effect and revealed the complexity of energetics in cancer cells as well as the metabolic heterogeneity of tumors [15,16,17]. The insight that the Warburg effect does not arise from defective mitochondria is not new [18]. It is widely understood to be a consequence of oncogenic signaling and hypoxic conditions. Still, we are only beginning to understand the role that metabolic dynamics play in the chemoresistance, metastasis and aggressiveness of CRC [19,20].

The deregulation of cellular energetics is considered one of the emerging hallmarks of cancer, although it is not yet fully understood to which degree these alterations are a consequence of proliferation-inducing oncogenes rather than a fundamental characteristic of cancer cells [12].

### 2.1. The Altered Energy Metabolism in CRC

The metabolic phenotype of a cancer cell can be attributed to several aspects of the malignant transformation including adaptions to the TME and mutations of metabolic enzymes. It can also occur as a consequence of oncogenic signaling, thus enabling an increased cellular growth rate and the provisioning of substrates for biosynthetic pathways. These observations led to the concept of metabolic reprogramming: the cancer-associated alterations in metabolism are not a passive response to damaged mitochondria, cell proliferation or survival signals but are a consequence of oncogene-directed metabolic reprogramming [21]. Furthermore, metabolites of the TCA cycle such as α-ketoglutarate, succinate and fumarate can participate in oncogenic signaling [22].

A growing tumor soon exceeds the size at which diffusion provides a sufficient oxygen supply, leading to hypoxic conditions in the tumor and a shift towards glycolysis. This shift is orchestrated by the hypoxia-inducible transcription factor 1α (HIF1α) which leads to the expression of several genes associated with the avoidance of hypoxic stress (e.g., glycolytic enzymes and glucose transporter *GLUT1*) and inhibits mitochondrial metabolism by activating pyruvate dehydrogenase kinase (PDK) [23,24]. Additionally, HIF1α stimulates the formation of new blood vessels that supply the tumor with oxygen and nutrients by inducing the expression of the angiogenetic growth factors VEGF and PDGFβ [25,26]. In CRC, HIF1α expression correlates with cancer-specific mortality and reoccurrence as well as vascular invasion [27]. Genes regulated by HIF1α were observed to be associated with chemoresistance in CRC. The induction of HIF-1α hydroxylation, catalyzed by prolyl hydroxylase domain protein (PHD) [28], and its subsequent protein degradation by zebularine, led to the reversal of oxaliplatin resistance and the inhibition of angiogenesis in an azoxymethane (AOM)/ dextran sodium sulfate (DSS)-induced CRC mouse model [19]. The mitochondrial pyruvate carrier (MPC), consisting of the subunits MPC1 and MPC2, was shown to be another crucial factor for the metabolic fate in CRC. As the MPC is responsible for the mitochondrial uptake of pyruvate, it enables its subsequent oxidation in the tricarboxylic acid (TCA) cycle [29]. The deletion or downregulation of *MPC1* and *MPC2* was observed in both colorectal tissue and cell lines and correlated with poor prognosis [30]. The expression of MPC led to an abrogation of the Warburg effect and re-established the oxidative metabolism in CRC cells, while impairing growth in mouse xenograft assays and the expression of stemness markers. Growth in standard adherent cell culture conditions was unaffected [30].

At the same time, a number of studies underline the role of OXPHOS in CRC. A functional comparative analysis of CRC biopsy material and the surrounding healthy colon tissue revealed a nearly unchanged glycolytic activity and an upregulation of OXPHOS in CRC cells [31]. In patient-derived microsatellite-stable (MSS) CRC tissue samples, an increased copy number of mitochondrial DNA (mtDNA) was observed, particularly in stage I and II cancers [32]. An increased mtDNA copy number in MSS CRC cell lines was shown to be associated with a higher proliferation and inhibition of apoptosis, caused by an induction of mitochondrial OXPHOS [33]. OXPHOS was also shown to be associated with the development of chemoresistance. The upregulation of OXPHOS occurred in the liver metastases of patients with CRC after chemotherapy with oxaliplatin and 5-FU and was linked to the development of chemoresistance. The chemotherapeutic treatment of patient-derived colonosphere cultures led to an increase in mitochondrial biomass and the expression of respiratory chain enzymes as well as higher rates of oxygen consumption mediated by the histone deacetylase sirtuin-1 (SIRT1) and its substrate, the transcriptional coactivator PGC1α [34]. Resistance towards 5-FU in CRC cell lines was associated with a metabolic shift towards OXPHOS. The resistant cells exhibited stem-like features and showed a high sensitivity towards the OXPHOS inhibitor metformin in combination with 5-FU [35]. In oncogene-addicted cancer cells, metabolic reprogramming to OXPHOS was observed to be involved in the mechanism of chemoresistance towards targeted therapy with the EGFR inhibitor gefitinib and the BRAF inhibitor vemurafenib in vitro [36].

An explanation for the contradictory results regarding the metabolic status of CRC may be the important role of oncogenes and mutated tumor suppressors. An investigation of the mtDNA copy number in healthy adenoma and carcinoma tissue of CRC patients revealed a decrease in malignant tumors. The mtDNA copy number was shown to be significantly lower in *BRAF*-mutated and microsatellite-instable (MSI) tumors and higher in *KRAS*-mutated tumors [37]. In accordance with this, the functional examination of the adenosine diphosphate (ADP)-activated respiration rate (Vmax) and the mitochondrial outer membrane (MOM) permeability in patient-derived *KRAS*-/*BRAF*-mutated as well as wild-type tumor specimen indicates that *KRAS* mutations might be linked to an oxidative phenotype, while *BRAF* mutations to a glycolytic phenotype [38]. This observation may contradict the findings in another study that revealed the induction of glycolysis, the accumulation of lactic acid and the sensitivity to glycolytic inhibition in *KRAS*-mutated CRC cells, whereas *BRAF*-mutated CRC cells remained resistant towards the alterations of glucose supply or metabolic inhibitors [39].

### 2.2. The Metabolic Impact of Oncogenes and Tumor Suppressors

Despite the initial belief that metabolic reprogramming is the result of the cellular adaptation response to unfunctional or damaged mitochondria, research in recent decades revealed the complex interaction between metabolic reprogramming and oncogene-mediated tumorigenesis. For metabolic reprogramming in the context of CRC, the most critical oncogenes are *MYC* together with *HIF*, the PI3K/AKT/mTOR axis and *KRAS* as well as *BRAF* with their interconnected, associated signaling pathways and the tumor suppressor *p53*.

#### 2.2.1. KRAS and BRAF

The small GTPase RAS as part of the MAPK/ERK pathway regulates cell proliferation, differentiation, adhesion, migration and apoptosis. About 40% of CRC tumors carry a *KRAS* mutation and these tumors are particularly difficult to challenge with therapeutic intervention using anti-EGFR antibodies, thus being associated with poor prognosis [40]. Mutually exclusive to *KRAS*, oncogenic *BRAF* mutations occur in less than 10% of CRC tumors, of which the most common type of mutation is *BRAF*-V600E [40].

Basically, *KRAS*-driven metabolic rewiring is tightly linked to the MAPK- and PI3K-pathway and their paralleled regulation of *HIF1α* [41]. Besides elevated basal macroautophagy, *KRAS* mutation leads to the reprogramming of cancer cell metabolism. One of the most common *KRAS* mutations, *KRAS*-G13D, drives glucose uptake by increased expression of GLUT1 next to enhanced lactate production, thereby fostering the Warburg effect as shown in the established isogenic CRC cell lines RKO wild-type/*BRAF*-V600E and DLD-1 wild-type/*KRAS*-G13D [42,43,44,45,46]. As shown in Caco-2 cells harboring *KRAS*-G12V, the glycolytic flux is upregulated [39]. In addition to the increased nutrient uptake via macropinocytosis and recycling, enhanced glycolysis is accompanied by the increased nonoxidative pentose phosphate pathway (PPP) and hexosamine biosynthetic pathway, fatty acid and nucleotide synthesis as well as phosphoserine biosynthetic pathway [42,47]. Especially for *KRAS*-mutated cell lines, glutamine dependency was shown [48,49]. Generally, the required glutamine is fueled into the TCA cycle as a carbon source and into anabolic biosynthetic pathways such as amino acid, nucleotide and glutathione synthesis as the nitrogen source [48,49]. *KRAS* enables cells to scavenge extracellular glutamine and to replenish anaplerotic pathways. Furthermore, the increased expression of enzymes within the glutamine metabolism were recorded (e.g., SLC1A5, GLS, GLUD1/2, GOT2) in CRC cell lines [42]. Particularly in human CRC tissue, the upregulation of the glutamine transporters SLC25A22 and SLC24A13 as well as an upregulation of the asparagine synthetase were detected [50,51,52]. However, glutamine dependency could not be shown in isogenic HCT116 and DLD-1 CRC for wild-type/*KRAS*-G13D cells by other research groups [43]. Despite these alterations, the mitochondrial function and the TCA cycle as well as OXPHOS are unaffected in the aforementioned CRC cell lines [43]. Mitochondrial OXPHOS is increased upon oncogenic *KRAS* mutation linked to HIF activation in vitro [53].

Much like *KRAS*, mutated *BRAF* was found to be associated with an altered energy metabolism in CRC. Isogenic RKO cell lines for wild-type/*BRAF* showed a Warburg phenotype with an increased expression of GLUT1 next to increased lactate production and significant changes to proteins of the glycolysis, nonoxidative PPP, glutaminolysis and the phosphoserine biosynthetic pathway [42]. Others found the glycolytic flux to be downregulated in isogenic CaCo-2 cells for *BRAF*-V600E [39].

#### 2.2.2. p53

In CRC, more than 60% of tumors bear a mutation in the tumor suppressor *p53*, leading either to a loss or gain of function and a loss of heterozygosity at chromosome 17p [54,55]. Mostly known for its crucial role in the regulation of the DNA damage response and cell cycle progression, mutated p53 was recently shown to alter energy metabolism in a variety of studies.

Orchestrating metabolism, wild-type p53 targets two central carbon flows. Glycolysis is suppressed by wild-type p53 via the downregulation or direct binding of hexokinase 2 (HK2), phosphoglycerate mutase 1 [56,57,58] in vitro and in vivo and glucose-6-phosphate dehydrogenase (G6PD) [59] and via reducing the expression of various glucose transporters (*GLUT1*, *GLUT3*, *GLUT4*) [60,61,62] and their plasma membrane translocation [63]. Furthermore, the extracellular transport of lactate as a glycolytic product is inhibited by p53, since it suppresses the transcription of monocarboxylate transporter 1 (MCT1) in the cell culture and mouse models [64]. In contrast, *TIGAR* [65,66,67] and PFKFB3 as well as PFKFB4 are induced by p53, which reduce the intracellular levels of fructose-2,6-bisphosphate and, thus, stimulate glycolysis. The loss of functional p53 leads to enhanced glycolysis with increased lactate production, as demonstrated in isogenic HCT116 cells wild-type/p53-null [68]. On the other hand, p53 under physiological conditions promotes OXPHOS via the inactivation of PDK2 in vitro and in vivo [69] as well as the increased expression in Parkin in vitro [70] and the concomitant upregulation of PDC activity. In addition, p53 is responsible for elevation in *SCO2* (synthesis of cytochrome C oxidase 2) in CRC cell lines [71,72], an essential factor for the electron transport chain. Moreover, anaplerotic glutamine reverting into the TCA cycle is fostered by p53 through glutaminase 2 (Gls2) in vitro [73,74]. In contrast, mutant p53 correlated with increased PDK2 as compared to wild-type p53 in CRC patients [75].

#### 2.2.3. PI3K–AKT–mTOR Network

Phosphatidylinositol 3-kinases (PI3Ks) are a family of enzymes, which are important for intracellular signal transduction. They are activated by receptor tyrosine kinases (RTKs) and G protein-coupled receptors after binding growth factors like epidermal growth factor (EGF) or by oncogenes like *RAS* [76,77]. While growth factors interact with the regulatory subunit p85, RAS directly activates the catalytic subunit p110 of PI3K [77]. Active PI3Ks then phosphorylate phosphatidylinositol-4,5-bisphosphate (PIP2) into phosphatidyl-inositol-3,4,5-trisphosphate (PIP3). This second messenger activates downstream effectors like the serine/threonine kinase AKT and mammalian target of rapamycin (mTOR), which initiates metabolic programs to promote cell growth [78].

The activation of AKT can elevate ATP levels by increasing glycolysis and OXPHOS through the expression and membrane translocation of GLUT 1 [79,80]. By phosphorylating phosphofructokinase-2 (PFK2), AKT also activates PFK1, the rate-controlling enzyme of glycolysis [77]. Furthermore, AKT promotes the expression and activity of the glycolytic enzyme HK2 [81] by phosphorylation as well as the subsequent translocation to the MOM, and impedes apoptosis by inhibiting the release of cytochrome c [77].

Following its activation by AKT, mTOR becomes part of the mTOR complex (mTORC) 1 or mTORC2 [82]. While mTORC2 phosphorylates AKT for its full activation, mTORC1 balances the cell metabolism between anabolism and catabolism [82,83]. It promotes the protein synthesis by phosphorylating p70S6 Kinase 1 (S6K1) and eIF4E binding protein (4EBP). S6K1 induces mRNA translation by activating substrates like eIF4B. Simultaneously, mTORC1 phosphorylates and thus inactivates the eIF4B inhibitor-programmed cell death protein (PDCD4). Phosphorylated 4EBP dissociates from eIF4E, which enables the 5’cap-dependent mRNA translation [82]. Additionally, mTORC1 also promotes nucleotide synthesis by increasing the ATF4-dependent expression of methylenetetrahydrofolate dehydrogenase 2 (MTHFD2), an important enzyme of the tetrahydrofolate cycle that yields one carbon units for purine synthesis [82]. mTORC1 can also affect lipid synthesis by activating the sterol responsive element binding protein (SREBP) either through a S6K1-dependent mechanism or by phosphorylating the SREBP inhibitor Lipin1 [84,85]. Furthermore, the activation of SREBP by mTORC1 increases the flux through the PPP, which generates NADPH and other metabolites for an anabolic metabolism [82]. Together with the increased translation of HIF1α, this shunts the metabolism from OXPHOS to glycolysis [82,84].

Genetic alterations of the PI3K pathway are commonly found in CRC, which lead to a hyperactivation of PI3K and its downstream effectors, thereby supporting tumor growth [77,86]. Hyperactivation can be triggered by the overexpression of insulin-like growth factor 2 (IGF2) and IRS2 [86], or by the genetic deletion of phosphatase and tensin homolog (PTEN), a negative regulator of the PI3K pathway that dephosphorylates PIP3 [77]. A loss of PTEN was reported in 4% of non-hypermutated CRC samples [86] and in about 12% of CRC primaries [87]. Mutated PIK3CA, which encodes the catalytic subunit p110α, were found in 11–32% of CRC samples [88,89,90]. This does not only cause an increased expression of p110α, but also an AKT-independent upregulation of the glutamate pyruvate transaminase 2 (GPT2), which converts glutamine into α-ketoglutarate (αKG). This renders the cells more dependent on glutamine and supplements the TCA cycle [91].

#### 2.2.4. MYC

Myc is an oncogene that is aberrantly regulated in different cancers via multiple mechanisms [92,93]. In CRC, the increased transcription factor-dependent upregulation of Myc is caused either by activating *β-catenin* mutations or by inactivating *APC* mutations [94]. Furthermore, abnormalities of the PI3K–AKT–mTOR network as well as aberrant activities of growth factors and receptor tyrosine kinases (RTKs), can lead to an overexpression of Myc [95]. The oncogenic long non-coding RNA (lncRNA) GLCC1 can stabilize Myc by modulating interactions between Myc and the heat shock protein 90 (HSP90) [96]. By inducing the transcriptional upregulation of the microRNA (miRNA) miR-181d, which represses CRY2 and FBXL3, Myc is also stabilized in CRC [97]. Myc regulates the transcription of 15% of genes in the genome by using several mechanisms, such as the recruitment of DNA methyltransferase, to repress genes like *p21Cip* [98] and histone acetyl transferases [93,99].

Myc was found upregulated throughout all the stages of CRC [95], which were associated with the increased expression of up to 231 genes encoding for enzymes and transporters, which are involved in 346 metabolic reactions [95] like glycolysis, glutaminolysis and one carbon metabolism [95,100]. Myc does also have an impact on the cell cycle, tumorigenesis [101], mitochondrial biogenesis [95,102] and miRNAs [97]. Myc modulates the metabolism in CRC cells by upregulating anabolic genes involved in fatty acid synthesis (e.g., *PTT1*), de novo purine (e.g., *PRPS2*) and pyrimidine (e.g., *CAD*) synthesis as well as by downregulating catabolic genes like *CPT2* engaged in fatty acid oxidation [95]. Furthermore, Myc promotes glycolysis by upregulating glycolytic genes, such as *LDHA* [96], and by downregulating genes of the TCA cycle (e.g. *IDH3A*) [95]. Besides these effects on the metabolism, Myc also influences tumorigenesis and cell cycle progression. Myc regulates long non-coding RNAs (lncRNAs) like MYCLo 2, which were found overexpressed in CRC cells and tissues. MYCLo 2 can transcriptionally repress *CDKN1A* (p21), which favors tumorigenesis and prevents p21-dependent cell cycle arrest [101]. By downregulating miR-23, which suppresses glutaminase (GLS), Myc can upregulate GLS and enhance glutamine metabolism in cancer cells [103]. The activation of GLS1 and of the ASC amino acid transporter 2 (ASCT2) drives glutaminolysis in CRC cells [104].

The observed metabolic alterations suggest that the overexpression of Myc induces the Warburg effect and promotes cell growth [95], while highly expressed genes and transporters of one carbon metabolism seem to drive oncogenesis in a Myc-dependent manner [95,105].

#### 2.2.5. HIF

The transcription factor HIF1 is a heterodimer consisting of HIF-1α and HIF-1β subunits. While HIF-1β is constitutively expressed, HIF-1α expression is controlled by the cellular oxygen state [106,107]. Under normoxic conditions, the enzyme PHD hydroxylates HIF-1α at two proline residues, which leads to a ubiquitination and its degradation by the 26S proteasome [107,108]. During hypoxia, the PHD activity is downregulated and therefore HIF-1α is stabilized [107]. HIF-1 can also be regulated in an oxygen-independent manner, e.g., by growth factors that activate the PI3K pathway and thus stimulate HIF-1α synthesis via mTOR [109].

Upregulated HIF-1α expression was found in up to 55% of CRC tissue biopsies and correlates with tumor stage. The overexpression of HIF-1α was most prominent in stage III and IV CRC as well as in the liver and lymph nodes metastases [110]. HIF-1α regulates genes like *COX2*, *VEGF* and *GLUT1* by binding to their hypoxia-responsive element promotor regions [107]. Increased COX2 expression leads to enhanced prostaglandin E2 (PEG2) levels [111]. Together with the transcriptional upregulation of *VGEF*, HIF-1α induces vascularization and angiogenesis in CRC cells [111,112]. Another target gene of HIF-1α is *GLUT1*, whose expression can be increased by different mechanisms such as the upregulation of miR-424 [107]. Furthermore, HIF-1α stimulates the multidrug resistant protein (MDR 1/P-glycoprotein) in CRC cells and tissue, which is one of the main reasons for resistance to chemotherapy [113,114].

In conclusion, HIF-1α induces the transcription of many different genes, which are important for CRC development. Besides other functions, HIF-1α influences the glucose uptake [107], drives vascularization and angiogenesis [111,112] as well as the resistance against chemotherapeutics in CRC cells and tissue [113,114].

### 2.3. The Central Role of the Tricarboxylic Acid Cycle

The tricarboxylic acid (TCA) cycle occupies a central role in the cellular metabolism and functions as a hub for catabolic and anabolic processes. Acetyl-CoA derived from pyruvate, fatty acid or amino acid oxidation represents the main fuel source of the TCA cycle.

In the first step of the TCA cycle, the acetyl group derived from acetyl-CoA is transferred onto oxaloacetate to form citrate, a six-carbon compound. In the subsequent two steps of oxidative decarboxylation, αKG and then succinyl-CoA are formed while two molecules of CO_2_ and NADH are generated. During the reaction of succinyl-CoA to succinate, GTP is created and utilized to generate ATP. Afterwards, the enzyme succinate dehydrogenase, which is part of the electron transport chain, converts succinate into fumarate and generates two molecules of flavin adenine dinucleotide (FADH), malate and finally oxaloacetate, which are formed to continue the cycle. The TCA cycle increases the energetic yield per glucose molecule by further breaking down the pyruvate generated during glycolysis and providing the reduction equivalents NADH and FADH_2_ for OXPHOS [115].

**Figure 2:**
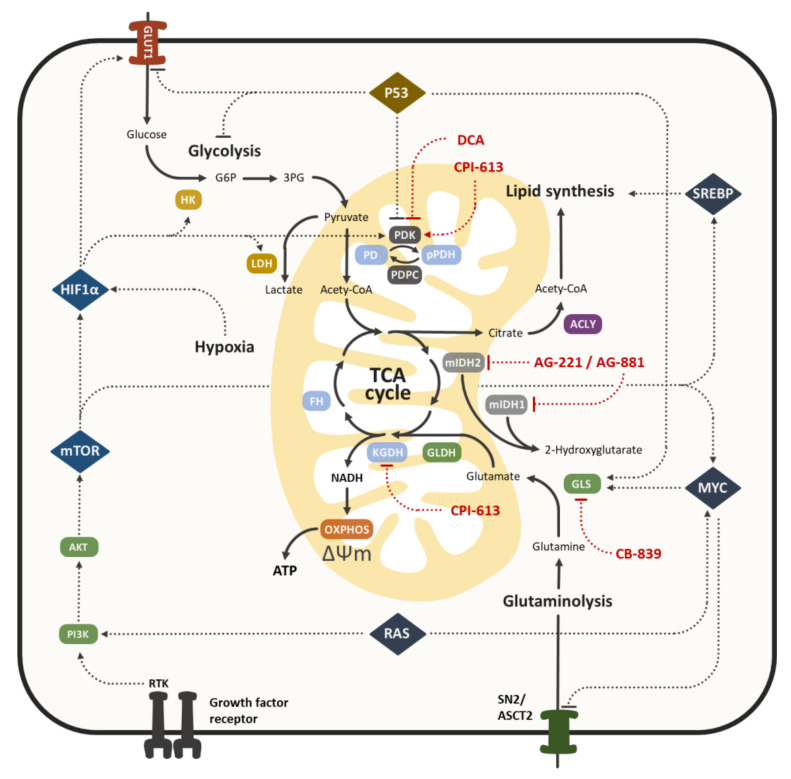
Oncogenic signaling and tumor suppressor activity in CRC cell metabolism. The metabolic pathways, centered around the TCA cycle, are indicated by continuous lines and include catabolic (glycolysis, OXPHOS), anabolic (lipid synthesis) and anaplerotic (glutaminolysis) reactions. Tumor suppressors (e.g., p53), oncogenes (e.g., RAS, MYC) and transcription factors (e.g., HIF1) regulate metabolic reprogramming by controlling the expression and activity of metabolic enzymes (e.g., HK, PDK, GLS) and transporters (e.g., GLUT1, SN2). Modulators of the TCA cycle (denoted in red), which are currently tested as chemotherapeutics in clinical trials, target different enzymes of the tumor cell metabolism (see chapter 3 for further details).

Since the numerous alterations involved in the aerobic glycolysis of cancer cells are very distinct, the role of the TCA cycle for cancer cell metabolism was used to be overlooked. The TCA cycle is not only the target of oncogenic signaling, thus enabling cancer cells to exploit this pathway for anabolic reactions, but also contains several intermediates that act as signaling molecules themselves, controlling cellular processes like DNA methylation, chromatin modification and hypoxic response [22]. Furthermore, enzymes of the TCA cycle frequently exhibit deregulated expression or mutations in cancer cells associated with metabolic alterations [116]. In the following section, we would like to illustrate some examples of the TCA cycle alterations observed in CRC (Figure 2).

Besides its importance for bioenergetics, the TCA cycle plays a central role in cellular anabolism. Intermediate molecules are shuttled from the mitochondrial matrix to the cytosol where they are utilized for the synthesis of fatty acids or non-essential amino acids. Studies utilizing a patient-derived xenograft model show that CRC cells rely on the TCA cycle as a source for their biosynthetic demands, including citrate for de novo lipid biosynthesis [117].

Since an excessive utilization of intermediates for biosynthetic purposes would lead to a breakdown of the TCA cycle, anaplerotic reactions are necessary to provide a steady refill [118]. Among others, the most important mechanisms are the generation of oxaloacetate by pyruvate decarboxylase utilizing cytosolic pyruvate and glutaminolysis. During glutaminolysis, glutamine is deaminated to glutamate by GLS and finally converted to αKG by glutamate dehydrogenase (GLDH). The upregulation of both enzymes was observed in patient-derived CRC samples, correlating with poor prognosis and tumor aggressiveness [119,120,121]. The dependence of CRC cells on glutamine for TCA cycle anaplerosis was shown in vivo, especially in tumors with an oncogenic mutation of the *PIK3CA* gene [91,117]. Other types of cancer, including non-small cell lung carcinoma and glioblastoma, use pyruvate anaplerosis by the enzyme pyruvate carboxylase to provide oxaloacetate for the TCA cycle [122].

Metabolite profiling of colon cancer tissue revealed a lower amount of metabolites involved in the TCA cycle compared to normal colon tissue and an increase in purines, pyrimidines and amino acids [123]. A reduction of TCA cycle-associated metabolites, namely fumarate, malate, succinate and oxalate, was observed in CRC tissue and accompanied by an increase in amino acids and metabolites associated with fatty acid synthesis [124,125]. A recent study showed that the inhibition of glycolysis led to increased OXPHOS in different tumor cell lines in vitro, and was accompanied by a decrease in TCA cycle intermediates and an induction of TCA cycle enzymes [126].

Mutations of the different enzymes involved in the TCA cycle are frequently observed in several types of cancer. Among the most affected enzymes is the isocitrate dehydrogenase (IDH) which can be subdivided into three isoforms: IDH1 can be found in the cytoplasm and peroxisomes, whereas IDH2 and IDH3 are located in the mitochondrial matrix. All three isoforms catalyze the irreversible, NAD-dependent reaction of isocitrate to αKG, while IDH1/2 isoforms also facilitate the reductive carboxylation of αKG to isocitrate, which oxidizes NADPH to NADP^+^ [127]. Mutations of a single arginine in the catalytic site of IDH1 and IDH2 were observed in several malignancies, including glioblastoma, acute myeloid leukemia (AML) and cholangiocarcinoma, leading to a gain-of-function. The mutated IDH catalyzes the conversion of αKG to the tumor-specific metabolite 2-hydroxyglutarate (2-HG), which induces cellular proliferation by activating the mTOR-signaling pathway and inhibiting αKG-dependent dioxygenases that are involved in the regulation of epigenetics and differentiation in vitro [116,128,129].

In an analysis of 152 CRC patients with a deficient DNA mismatch repair system (dMMR) and MSI, no mutations of IDH isoenzymes 1 and 2 were observed, including sporadic dMMR CRC with a hypermethylation of the *MLH1* gene promoter that is associated with the CpG island methylator phenotype (CIMP) [130]. In contrast, the examination of potential IDH1 alterations in 161 MSS CRCs revealed four cancers with IDH1 mutation, which were all CIMP-positive and *BRAF* mutant [131]. This led to the assumption that 2-HG produced by mutant IDH1 can induce CIMP in a small proportion of *BRAF*-mutant MSS CRCs, a mechanism that was observed in glioblastoma and is associated with poor prognosis [132]. Molecular profiling of 428 patients with advanced CRC revealed a IDH1 mutation rate of 0.9% [133]. More recent studies observed IDH1 mutations in 7% of inflammatory bowel disease-associated (IBD) CRC [134] and 11% of colitis-associated CRC [135], compared to 1% of sporadic CRC, providing a therapeutic target in these types of CRC tumors.

The levels of 2-HG were found to be elevated in the absence of IDH mutations in human CRC specimen and cancer cell lines HCT116 and RKO, and were shown to be associated with epithelial–mesenchymal transition (EMT) and distant metastases. A possible mechanism is the trimethylation of histone H3K4 in the promoter region of ZEB1, presumably due to an inhibition of histone deacetylases by 2-HG. The elevated glutamine anaplerosis in CRC was assumed to be a driving factor for increased 2-HG levels [136].

In a recent study, the role of αKG as a signaling molecule in CRC was elucidated: the hypomethylation of DNA and histone H3K4me3, induced by αKG, led to an upregulation of differentiation-associated genes and a downregulation of Wnt target genes in an intestinal organoid model. The restriction of glutamine results in reduced αKG levels, which subsequently activates the Wnt pathway and promotes cancer stemness [137].

### 2.4. Metabolic Symbiosis in the Tumor Microenvironment

The diversity in a tumor has three main drivers: The genomic instability of cancer cells leads to the generation of subclones with distinct phenotypes, the differentiation of cancer stem cells (CSCs) results in cellular heterogeneity and the tumor microenvironment (TME) generates varying selective pressure in different regions of a tumor [138]. Thereof, solid tumors are composed of a variety of cell types, featuring not only morphologic, genetic and epigenetic differences but also specific metabolic adaptions. As mentioned previously, these adaptions can arise from intercellular oncogenic signaling as a consequence of certain conditions in the TME, including hypoxia and nutrient deprivation. Besides malignant cells, the TME is also composed of blood vessels, cancer-associated fibroblasts (CAFs), immune cells and the extracellular matrix (ECM), intertwined by the exchange of metabolites and intermediates. In particular, CAFs were observed to interact extensively with cancer cells in a metabolic symbiosis, often referred to as the reverse Warburg effect. According to this model, aerobic glycolysis is induced in CAFs by the activation of HIF1 due to elevated reactive oxygen species (ROS) production in cancer cells. The accumulating lactate is shuttled as an energy source to nearby malignant cells where it is further metabolized in the TCA [139].

This interaction of cancer cells and CAFs was observed for several CRC cell lines and associated with a reduced expression of the TCA enzyme IDH2, the induction of the glycolytic enzyme PKM2 and GAPDH expression, which increased autophagy in CAFs mediated by ROS signaling [140]. In contrast to this theory, the functional analysis of CRC tumor specimens revealed not only OXPHOS-centered metabolism in cancer cells but also the induction of OXPHOS in nearby tissue cells together with an increased MOM permeability against adenine nucleotides [31]. Another variant of metabolic symbiosis was revealed by the immunohistochemical examination of colorectal carcinoma: cancer cells expressed transporters (GLUT1, MCT1) and enzymes (LDH5, HIF1) associated with anaerobic metabolism, whereas CAFs lacked these proteins and expressed transporters (MCT1, MCT2) and enzymes (LDH1) involved in lactate uptake and oxidation [141]. These studies suggest that the type of metabolic symbiosis in the TME of CRC might be just as diverse as cancer cell metabolism itself.

In CRC, cancer stem cells (CSCs) are an essential component of a solid tumor, even if they only represent a small proportion of the total cell population [142]. The ability to self-renew and produce differentiated daughter cells enables CSCs to facilitate tumor growth and self-sustained proliferation [143]. Tumor chemoresistance is associated with several characteristics of CSCs, including a slow cell turnover, the occupation of protective niches in the TME, the expression of drug efflux transporters and anti-apoptotic signaling. Thus, CSCs are often responsible for relapse after initial response to a chemotherapeutic treatment, which makes them a promising therapeutic target [144].

Different metabolic adaptions were observed in CSCs, with no clear dependence on the examined tumor entity: a shift towards a highly glycolytic phenotype may occur as well as a dependence on OXPHOS. Regardless of the metabolic preference, mitochondria were found to play an essential role, not only in CSC metabolism but also in the regulation of stemness and avoidance of apoptosis [145]. This metabolic ambiguity also applies for the CSCs of colorectal tumors. In CD133(+) Colo205 cells a higher expression of glycolysis and TCA cycle enzymes as well as a reduced ATP and glucose-dependent lipid synthesis was observed in vitro compared to CD133(-) cells [146]. Mitochondrial metabolism was shown to be downregulated in CRC by an inhibition of pyruvate import. The re-expression of the pyruvate transporters MCP1 and MCP2 was associated with impaired stem cell properties in vitro and in a mouse xenograft model [30]. In contrast, colorectal CD133(+) CD44(+) Lgr5(+) CSCs derived from human tumor specimen exhibited an induction of OXPHOS and a higher susceptibility to the blocking of FOXM1-dependent expression of the mitochondrial antioxidant enzyme peroxiredoxin-3 than non-CSCs [147]. The importance of glutamine for non-stem CRC cells was described before. In addition, colorectal CSCs were shown to rely on the glutamine pathway to a variable extent [148]. Targeting the metabolic characteristics and adaptions of CSCs may be a promising strategy to impede relapse after chemo- or radiotherapy [149]. Therefore, a better understanding of the metabolic reprogramming that occurs due to therapy is necessary in colorectal CSCs.

Moreover, several types of immune cells, such as T lymphocytes and tumor-associated macrophages (TAMs) are recruited by the release of cytokines or expand during tumor formation [17], like myeloid-derived suppressor cells. As the function, activation and differentiation of immune cells are regulated by their intracellular metabolism, the unique conditions of the TME can inhibit their anti-tumor activity through different mechanisms [150].

Competition over nutrients was found to be one of the components of intratumoral immunosuppression [151]: upon activation to effector T cells, naïve T cells exhibit a MYC-dependent [152,153] reduction of TCA cycle activity, increased aerobic glycolysis and a higher expression of GLUT1, leading to an excessive demand for nutrients [154,155], in particular glucose and glutamine. Due to the high nutrient demand of tumor cells, glucose availability for T_eff_ cells is limited, attenuating their effector activity [156]. Reduced levels of phosphoenolpyruvate, an intermediate of glycolysis functioning as a signaling molecule in T_eff_ cells, contributes to a decrease in T cell activity [157]. Additionally, T cell activity requires glutamine [158], an amino acid with a highly restricted availability in tumors due to a high demand in most cancer cells [117]. The nutrient deprivation that T cells are confronted with in a TME cannot only result in an abrogation of their tumoricidal effects, but also lead to the generation of dysfunctional exhausted T cells [159].

In contrast to T_eff_ cells, regulatory T cells are often associated with poor prognosis and restrict local anti-tumor immunity [160]. T_reg_ cells do not share the same requirement for glucose as T_eff_ cells and express low levels of GLUT1, but instead rely on OXPHOS and lipid oxidation, rendering them resistant against the nutrient shortages in the TME [161]. This phenotype is regulated by the transcription factor forkhead box protein P3 (FOXP3), by abolishing the PI3K/AKT/mTORC1 axis, down-regulating the genes involved in both glycolysis and glucose uptake and inducing lipid metabolism [162].

TAMs, which generally have a tumor-promoting activity and facilitate metastasis [163,164] and angiogenesis, mainly rely on glycolysis. The inhibition of HK2 using 2DG led to an abrogation of the pro-tumorigenic activity in vitro [165]. In macrophages, the induction of glycolysis is necessary for inflammatory signaling and mediated by elevated levels of the TCA cycle intermediate succinate and subsequent induction of HIF1α [166]. In a recent study, the secretion of succinate into the TME by tumor cells was observed, which led to the polarization of macrophages to TAM [167], mediated by the PI3K–HIF1α axis.

Compared to many essential nutrients, which are scarce in the TME, byproducts of the tumor cell metabolism can be found in abundance, with lactate being the most important one [168]. In general, the impact of lactate on immune cells may be subdivided into specific molecular effects and the consequence of an acidification of the TME: lactic acid induces the polarization of TAM, characterized by VEGF and arginase 1 expression, and this process was mediated by HIF1α in vitro and in vivo [169]. The direct lactylation of histones that regulates gene transcription from chromatin was recently observed in macrophages [170]. The effects of low pH on the function of immune cells are well described and include decreased cytokine production [171], a decline in T cell responsiveness [172] and the impaired proliferation and activation of T cells [173].

## 3. Cancer Specific Mitochondrial Metabolism as a Therapeutic Target

Considering the diverse side effects of traditional chemotherapy, the discovery and exploitation of cancer vulnerabilities may lead to the development of more efficient as well as more tolerable chemotherapeutic treatment [109,174,175,176]. In recent years, a continuous increase in knowledge about cancer-specific metabolism provided new targets for the development of small molecule therapeutics, some of which are currently used in clinical trials (Table 1). At the same time, the complexity and adaptability of tumorigenic metabolism may result in unforeseen consequences of a therapeutic intervention. First attempts to target cancer metabolism aimed at the aerobic glycolysis as the most obvious alteration in tumor cells, while recent research focuses on the TCA cycle and OXPHOS [177,178]. A vast number of drugs under investigation are targeting the mitochondria and their function, e.g., metformin, 3-bromopyruvate or 2-deoxyglucose [179,180]. Among these, the antidiabetic drug metformin modulates mitochondrial metabolism and shows a pleiotropic mode of action interacting with several metabolic and non-metabolic signaling pathways [181,182]. Primarily, energy-sensing pathways (e.g., AMPK) and other targets are activated and mitochondrial complex I is inhibited, upon which mitochondrial respiration is decreased and aerobic glycolysis is increased [182,183]. Metabolism through the TCA cycle and OXPHOS are blocked, which starves cancer cells and based on this observation, metformin is described as drug reducing the risk for the development of cancer [183,184]. Metformin was shown to have anticancer effects in cell culture and mouse models of CRC as well as in epidemiological studies and clinical trials (reviewed in [184]). Notwithstanding, in tumor cells, the TCA cycle serves as the main hub for anabolic and catabolic reactions and can be dysregulated due to oncogenic signaling as well as up/downregulated or mutated enzymes [185]. Therefore, different enzymes of the TCA cycle may serve as a promising target for the development of novel tumor therapeutics, either as a single-drug or in combination with a cytostatic agent [186].

Dichloroacetate (DCA) indirectly targets the aerobic glycolysis by facilitating the entry of pyruvate into the TCA cycle: It inhibits the enzyme PDK, inducing higher levels of active PDC, which can be depleted in tumor cells due to HIF-signaling. Active PDC converts pyruvate to acetyl-CoA, thereby attenuating tumorigenic lactate production [187]. DCA for the treatment of hematological malignancies, head and neck carcinomas, glioblastoma, breast cancer and lung cancer went into clinical trials in late 2007 but could not reach Phase III and was disregarded a few years later. In a recent study, the promising effects of DCA in combination with 5-FU were observed in chemoresistant CRC. DCA led to a p53-dependent expression of miR-149 and miR-149-3p, thereby attenuating PDK2 activity and abrogating the Warburg effect [75].

Several inhibitors of mutated IDH 1 and 2 are currently under development and show a promising antitumorigenic potential in preliminary trials. By selectively inhibiting the activity of mutated IDH 1 and 2, the accumulation of the oncometabolite 2-HG is prevented. This abrogates the detrimental effects of 2-HG signaling and corresponding feedback mechanisms in IDH 1/2 mutated tumors, leading to an improved survival rate [188]. The mutant IDH 2 specific inhibitor AG-221 (Enasidenib^®^) is currently being tested for the treatment of hematological malignancies including AML in several trials. A Phase I/II trial for the usage in solid tumors with IDH 2 mutation was completed 3 years ago, but the results have not been published (NCT02273739). The mutant IDH 1 and IDH2 inhibitor AG-881, which is able to pass the blood–brain barrier, is currently used as a standalone drug in clinical trials to treat IDH-mutated glioma patients (NCT02481154, NCT04164901, NCT03343197), and was used in a Phase I study in patients with hematological malignancies (AML, myelodysplastic syndrome) already completed (NCT02492737). To summarize, more than 20 clinical trials included the AG-221 treatment of cancer patients with AML, myelodysplastic syndrome or other types of hematopoietic malignancies since 2013, of which some moved into Phase III. In 2014, the FDA granted orphan drug status for AG-221 for AML, and European Medicines Agency (EMA) followed in 2019. A handful of studies with AG-881 for treating glioblastomas and hematopoietic malignancies peaked with a Phase III trial in glioblastoma patients in the beginning of 2020. In the future, IDH inhibitors might provide an additional therapeutic option for the treatment of IBD-associated CRC, which exhibit a IDH 1 mutation rate of 7% (IBD-associated CRC) and 11% (colitis-associated CRC), respectively [134,135].

The development of the specific and potent GLS inhibitor CB-839 enabled a targeting of the TCA cycle by stopping anaplerosis via glutamate. Tumor cells utilize glutamine to synthesize αKG via the enzymes GLS and GLDH, since TCA cycle intermediates are increasingly used for biosynthesis [189]. First Phase I trials regarding the applicability of CB-839 for the treatment of solid tumors and hematological malignancies have been completed and more studies, including the treatment of CRC in combination with Capecitabine, are currently recruiting (NCT02861300). To date, CB-839 was tested in about 20 studies in a variety of solid tumors next to hematopoietic malignancy conditions, but none of the study designs to date have reached Phase III. However, the FDA allowed the fast track designation of CB-839 for metastatic renal cell carcinoma in 2017. The combinatory application of CB-839 and the GLUT-1/-3 selective inhibitor Glutor led to the inhibition of cancer cell growth in HCT116 CRC cells, even under high glutamine and glucose conditions [190]. Therefore, synergistic effects of GLS inhibitors in combination with additional metabolic modulators might be the subject of future research. The recently developed CB-839 derivatives CPD-20 and CPD-23 exhibit a stronger inhibition of GLS and better tumor accumulation, resulting in improved antitumorigenic effects [191,192].

Based on the endogenous cofactor α-lipoic acid (LA), the multipurpose metabolic inhibitor and LA derivative CPI-613 was developed. By inhibiting the enzyme complexes pyruvate dehydrogenase (PDC) and the α-ketoglutarate dehydrogenase (KGDH) complex, CPI-613 targets the deregulated TCA cycle of cancer cells [193,194]. The first clinical trials with CPI-613 were initiated in early 2008 and the conditions treated included both solid tumors as well as hematopoietic malignancies with a focus on advanced, metastatic or recurrent and refractory diseases in more than 20 relevant clinical trials. Several Phase II and two Phase III trials evaluated the safety and efficacy of CPI-613 until today and CPI-613 was granted orphan drug status for conditions such as AML and pancreatic cancer by the FDA and EMA. In the following section, the antitumorigenic activity of CPI-613 will be discussed in detail.

## 4. The Lipoic Acid Derivative CPI-613 in Cancer Therapy

The endogenously occurring disulfide compound LA belongs to the group of lipoates and displays various anti-cancer features. These anti-tumorigenic activities of LA were extensively reviewed by our group and others elsewhere [195,196]. In short, LA possesses a high redox capacity due to its disulfide bond and forms a redox pair with its reduced form, dihydrolipoic acid. Therefore, LA displays anti-oxidative properties by directly scavenging ROS, the chelation of transition metal ions, the regeneration of antioxidants such as ascorbic acid and glutathione and by activating the transcription factor Nrf2 [195]. Additionally, LA acts anti-inflammatory via the blockage of the NF-κΒ pathway and inhibits invasion, migration and angiogenesis in tumor tissue [195]. Upon LA treatment, the p53-independent intrinsic mitochondrial pathway was elicited in CRC cells [197,198]. Other effects included the induction of (macro-) autophagy, the depletion of the direct DNA damage reversal protein *O*^6^-methylguanine DNA-methyltransferase (MGMT) and the tumor suppressor p53 in CRC cells [199,200].

Derived from LA, the structure of CPI-613 (6,8-bis(benzylsulfanyl)octanoic acid) does not contain a disulfide bond, but two benzyl rings that are covalently linked to the sulfur atoms [194]. Due to this structural alteration, CPI-613 has no potential to perform redox reactions and interferes with the physiological function of LA as a mitochondrial cofactor for the crucial multi-enzyme complexes involved in aerobic metabolism.

### 4.1. The Molecular Mechanism of CPI-613 Antitumor Activity

CPI-613 has been described as the first compound to selectively disrupt altered mitochondrial energy metabolism in cancer cells and forms a new distinct class of potential anticancer drugs. Thus, it is said to have a broad efficacy against a wide range of cancer entities. As detailed in two landmark studies, CPI-613 primarily targets two central multi-enzyme complexes in the mitochondrial TCA cycle as mechanistically demonstrated in cell culture experiments [193,194]. Under physiological conditions, LA is used as redox-cycling cofactor within the E2-subunit of the PDC complex and KGDH complex [201]. To fulfil this function, LA is covalently linked to the E2 subunit of PDC. By exploiting its redox inertness, CPI-613 as a derivative of LA inhibits both multi-enzyme complexes, which perform the oxidative decarboxylation of pyruvate to acetyl-CoA and α-KG to succinyl-CoA, respectively.

Nevertheless, the molecular mechanism of PDC and KGDH inhibition are distinct. KGDH activity is generally auto-regulated by the generation of ROS at the E3 subunit (dihydrolipoyl dehydrogenase) and mediated by the ROS-sensing E1 subunit (oxoglutarate dehydrogenase). Upon incubation with CPI-613 in vitro, a ROS burst originating from the E3 subunit interferes with the auto-regulation and KGDH is constitutively self-inactivated [193]. The inhibition further involves glutathionylation as well as redox blockage of the E2 subunit (dihydrolipoamide succinyltransferase) and leads to a perpetuation of the TCA cycle as well as an abrogation of glutamate or ammonia entry in the TCA cycle. Downstream metabolites such as succinate, fumarate and malate are deprived. Oxygen consumption is impaired at concentrations of 5–50 µM CPI-613 in leukemia cell lines, which, however, is not a sufficient concentration to inhibit OXPHOS in glioblastoma cell lines [202,203]. The addition of the antioxidant *N*-acetyl cysteine reverses the effect of CPI-613 in vitro [193]. On the other hand, CPI-613 inhibits PDC indirectly by interfering with its regulation via PDKs. PDKs are tumor-selectively-activated via phosphorylation at Ser360, Ser293 and Ser232 upon incubation with CPI-613, which in turn leads to an inhibitory hyper-phosphorylation of the E1α subunit (pyruvate dehydrogenase) of PDC [194]. Consistent with this finding, the knockdown of PDK recovers this effect. As explained earlier, PDC activity is tightly linked to oncogenic mutations. Although in some studies CPI-613 elicited a ROS burst, other research groups use CPI-613 as a ROS-minimizing agent in other models and conditions [204,205]. In general, CPI-613 is applied as either a PDC or KGDH inhibitor in yeast, cell culture or murine experiments [206,207,208,209,210]. It is important to note that metabolite and carbon source availability are mediating the cytotoxicity of CPI-613 in cell culture experiments [193,194]. For example, in low glucose or low serum conditions, the cytotoxicity of CPI-613 is enhanced due to these starving conditions and a shift towards OXPHOS [194]. Furthermore, carnosine, an inhibitor of glycolytic ATP production, and CPI-613 act synergistically by depriving all energy sources originating from glucose [203]. Primarily targeting the TCA cycle, CPI-613 has marginal effects on proteins of the glycolytic pathway, such as HK I and II, lactate dehydrogenase A (LDHA) and pyruvate kinase M2 (PKM2) in vitro [211]. Furthermore, CPI-613 was also shown to impact lipid metabolism mediated by the 5′ AMP-activated protein kinase (AMPK)–Acetyl-coenzyme A carboxylase (ACC) axis, as shown in CPI-613-treated pancreatic cancer cell lines with decreased lipid accumulation [211]. The disruption of carbon and energy flux as well as the perpetuation with biosynthetic intermediate supply by CPI-613 leads to selective cancer cell death in a variety of in vitro and in vivo models (see below).

The potential of CPI-613 to reduce cancer cell growth was initially shown in different cancer cell lines originating from colorectal, breast, lung, kidney, ovarian, prostate, pancreatic or bone marrow cancer with inhibitory concentration (50%) (IC_50_) values ranging between 100 and 280 µM [194]. By comparing to non-transformed cell lines, CPI-613 displays tumor selectivity. Additionally, treatment with CPI-613 leads to cell death also in p53-deficient or *KRAS*-mutated cell lines [194,212,213] as well as in drug-resistant cells [214,215,216]. Microarray data showed the reduced expression levels of cyclins as well as *CDK2*, *p27* and *p19*, which point to a preceding cell cycle arrest [217,218]. Upon treatment with CPI-613, apoptosis characterized by Caspase-3 and PARP1-cleavage, an increase in pro-apoptotic Bax or NOXA and a decrease in anti-apoptotic Bcl-2 as well as membrane blebbing were found in H460, LNCaP, AsPC-1 and PANC-1 cells [211]. However, the pan-caspase inhibitor zVAD was not able to reverse this effect [194]. Thus, multiple redundant pathways (e.g., necrosis) are triggered by CPI-613 [194]. Apoptosis was elicited in a ROS-dependent manner as shown using *N*-acetyl cysteine as an antioxidant in the pancreatic cancer cell lines AsPC-1 and PANC-1 [211]. Partially contradicting, Egawa et al. failed to detect apoptotic features, such as DNA fragmentation, in fibroblasts, which suggests a cell type-dependent mode of action [219]. Hence, more detailed studies on cell death mechanisms triggered by CPI-613 are required. As visualized using transmission electron microscopy, mitochondrial morphology was altered by CPI-613, showing reduced mitochondrial cristae junctions and overall disrupted cristae morphology in pancreatic cancer cell lines. These morphological changes occurred concomitantly to mitochondrial membrane dissipation [211]. Surprisingly, chronic treatment with CPI-613 does not reduce mitochondrial number and size, but only impairs mitochondrial membrane potential in cell culture models [194,220], which is also observed in an irreversible manner upon short-term treatment. Moreover, the CPI-613-mediated activation of AMPK was described in AML cells [212]. The activation of the AMPK–ACC axis was in turn observed to be crucial for the induction of apoptosis in pancreatic cancer cell lines [211]. Ambiguously, CPI-613 in combination with chloroquine, but not CPI-613 alone, caused autophagosome formation followed by lysosome fusion in clear cell sarcoma cells [219]. CPI-613 in combination with PS48 increased autolysosome formation in porcine fibroblasts, however without elevating LC3B levels [220]. However, Gao et al. recorded CPI-613 monotreatment to increase autophagy with an accumulation of LC3B-II, a decrease in p62 and an increase in double membraned vacuoles in pancreatic cancer cell lines via the AMPK–ULK1 pathway. The sum of these observations suggests a cell type-specific cellular response to the treatment with CPI-613. The elicited autophagy was, however, noted to be not influencing the apoptotic rates [211].

Although some mechanistic studies are available, the necessity for comprehensive studies in particular regarding cell death mechanisms upon CPI-613 treatment and the role of autophagy needs to be underlined.

### 4.2. Cancer Stem Cells as a Preferential Target of CPI-613?

As outlined earlier, CSCs possess a unique flexibility in metabolic adaption and are metabolically distinct to non-CSCs. Whereas normal stem cells primarily rely on OXPHOS and cancer cells utilize glycolysis, CSCs have the capability to quickly switch between metabolic pathways and symbiotically provide bioenergetic and biosynthetic intermediates, but principally lean on the TCA cycle. This feature predestines these cells to be vulnerable to treatment with CPI-613. Additionally, CSCs are found upon standard chemotherapy, are typically drug-resistant themselves and foster chemoresistance, and therefore, are crucial in the development of recurrent disease, e.g., recurrent platinum-resistant ovarian cancer [221]. Here, recurrent platinum-resistant disease is tightly associated with poor prognosis, and hence, is a therapeutic challenge [222]. Targeting CSCs with CPI-613 has thus been postulated to delay or prevent the development of recurrent (platinum-resistant) disease by Bellio and colleagues [223].

Addressing this hypothesis, CPI-613 treatment significantly decreased CSC frequency, expressed as CD133+/CD117+ cells, in five ovarian cancer cell lines and additionally impaired cell viability and sphere-forming capacity. In ovarian cancer, CSCs (CD133+/CD117+) were revealed to depend on OXPHOS instead of glycolysis, show concomitantly elevated ROS levels as well as mitochondrial membrane potential and have pro-tumorigenic features [224]. In vivo, CPI-613 abrogated the sphere-forming capacity and tumorigenicity of OVCAR3-derived xenografts in mice. Furthermore, CPI-613 (12.5 mg/kg BW/d; twice in 10 days) reduced CSC frequency with slight but significant tumor growth inhibition, also upon CSC induction by carboplatin/paclitaxel treatment. In another study [225], stem-like H460 cells expressing an EMT phenotype were eliminated almost as effectively as the parent H460 cells.

Solid tumors form a cooperative metabolic network with cancer-associated stroma, such as CAFs, in their microenvironment using the “reverse Warburg effect” as described beforehand. While CAFs play a crucial role in the development of metastasis and the mediation of chemoresistance, they are primed to predominantly use glucose as an energy source, produce lactate and pyruvate from glucose and exhibit augmented levels in (macro-)autophagy and mitophagy [139,226,227,228]. Therefore, CPI-613 should rather target the core of solid tumors instead of neighboring stromal cells. Albeit this theory, Mordhorst et al. observed a “reverse Warburg”-like effect with a change in more than 25 genes and 20 metabolites in cancer-associated stroma upon treatment with CPI-613 and PS48, an inhibitor of 3-phosphoinositide-dependent protein kinase-1, in porcine fetal-derived fibroblasts in vitro [229]. Overall, pyruvate and glutamine levels were elevated and regarded as a driver for amino acid and fatty acid synthesis.

In conclusion, CPI-613 was identified as a candidate drug to complement and improve current therapy strategies by overcoming unintended CSC enrichment with accompanied chemoresistance and by interfering with the metabolic coupling in a preclinical set-up. Nevertheless, the CSC-directed cytotoxicity of CPI-613 has not been proven for other cancer entities and tumor microenvironments in such detail yet and needs further elucidation. The described studies, likewise, lacked proof for a long-term benefit based on the suppression of the CSC population and the disturbance of metabolic symbiosis.

### 4.3. Application of CPI-613 in Preclinical Studies and Clinical Trials

Preclinical evaluation is, until now, limited to murine xenograft experiments with established cancer cell lines. Using H460 lung cancer cells, tumor growth was significantly reduced by CPI-613 (10 mg/kg BW), independently of the administration frequency [194]. A 50% reduction in tumor growth and a 4-fold increase in survival were observed upon a dose of 25 mg/kg BW CPI-613 in pancreatic BxPC3 xenografts, which was even superior to the first line drug gemcitabine [194]. Using positron emission tomography (PET) scanning, Sai et al. indirectly monitored the delivery of CPI-613 into BxPC3 xenografts [230]. A combination of 25 mg/kg BW CPI-613 and chloroquine (50 mg/kg BW) once per week abolished the spread of clear cell sarcoma cells to the mesenteries [219]. However, CPI-613 and chloroquine alone failed to show this anti-metastatic effect or at least a reduction in xenograft tumor growth [219]. None of the studies described reported severe side effects or hematologic alterations [194]. Very recently, a biodegradable copolymer of lysine- and PEG-based monomers has been developed to simultaneously deliver CPI-613 and LY2109761, a TGF-β receptor I/II and collagen I inhibitor [231]. Due to the targeted delivery, pancreatic cancer progression in an orthotopic mouse model was markedly reduced [231].

A total of 21 clinical trials evaluate the applicability and efficacy as well as safety and tolerability of CPI-613 in the treatment of cancer to date. A variety of Phase I, I/II, II and III studies focus on the applicability of CPI-613 either as a single agent or most frequently in a combination regimen with established chemotherapeutics in the treatment of diverse cancer entities (see above, Table 1). Among these are hematologic malignancies with a focus on AML and solid tumors, including advanced and metastatic CRC. In general, the FDA granted orphan drug status for CPI-613 with the brand name Devimistat^®^ for AML, pancreatic cancer, myelodysplastic syndrome, peripheral T-cell and Burkitt’s lymphoma. In 2018, the EMA followed to grant orphan drug status for AML and pancreatic cancer.

Once administered, CPI-613 undergoes biotransformation including phase I oxidation via CYP3A4/5 and CYP2C8 with partially coupled phase II *O*-glucuronidation and sulfoxide formation as shown in vitro using human S9 mix [214,215]. With respect to cytotoxicity, drug metabolism leads to a decrease or loss of cytotoxicity as compared to the parent compound. Regarding toxicokinetics, the maximum plasma concentration is about 40 µM upon the maximum tolerable dose (MTD) of 2940 mg/m^2^ and the half-life of CPI-613 is about 2 h with a triphasic elimination due to enterohepatic circulation [202,214,215,232].

Numerous studies assessed the inclusion of CPI-613 in standard chemotherapy as beneficial in terms of chemotherapy response rates, patient performance as well as overall and progression-free survival. Early clinical trials recorded response rates of 29% to CPI-613 monotherapy as compared to 80% for CPI-613 in combination with bendamustine in patients with AML and lymphoma of various types [202,233]. The response was tightly linked to upregulated T-cell activation and the regulation of cytokine production in peripheral blood mononuclear cells (IFNG, CCL5, CRCR3, CD8A, CD3E, CD3D). The overall MTD of CPI-613 was 2940 mg/m² when administered via the central vein to avoid inflammation in the peripheral vein [202]. In respect to relapsed or refractory AML, CPI-613 only increased the response rates in patients of advanced age and/or poor performance in a Phase I study in combination with high-dose cytarabine and mitoxantrone [212]. In younger patients, which did not respond to the therapy regimen, a significant increase in superoxide dismutase 2 was noted. The responsiveness of older patients was shown to be associated with immune system upregulation via the activation of B-cells by the overexpression of *CD79A*, *MS4A1*, *FCRL2*, *TCL1A*, *BANK1+*, and *CD19+*. This could be attributable to the low spare respiratory capacity and decreased mitochondrial quality, which is associated with advanced age. In the corresponding Phase I/II clinical trial with CPI-613 in combination with high-dose cytarabine and mitoxantrone, older patients had a 52% complete remission and a median survival of 12.4 months [234]. Pardee et al. assumed that, upon exposure to DNA damaging agents, the cells are in an enhanced need for energy-consuming DNA damage repair, and thus, the upregulation of the TCA cycle renders pre-treated tumors vulnerable to CPI-613 treatment. This trial series is further proceeded into a Phase III trial, named ARMADA2000.

In advanced or metastatic pancreatic cancer patients, CPI-613 displayed, alone or in combination with gemcitabine, an improvement of the overall survival as compared to paclitaxel, gemcitabine or FOLFIRINOX in a Phase I clinical trial [217]. In another Phase I trial with metastatic pancreatic cancer patients also including *KRAS* or *p53* mutations, CPI-613 was combined with a modified FOLFIRINOX regimen [232]. An overall partial or complete response rate of 61% was observed, which represents a doubling in comparison to the modified FOLFIRINOX alone. Furthermore, the inclusion of CPI-613 into the combination regimen did not augment the toxicity. A corresponding Phase III trial known as AVENGER500 was initiated [235].

Although CPI-613 was positively evaluated in clinical trials, a Phase II clinical trial in patients with refractory or relapsed small cell lung carcinoma was suspended early due to the lack of efficacy of CPI-613 monotherapy in 2016 [216]. Nevertheless, in the same study, the patients were found to be sensitized to the standard chemotherapeutic topotecan by the administration of CPI-613. This sensitizing effect was also detected in AML cell lines once treated with CPI-613 together with doxorubicin or cytarabine and in combination with the tyrosine kinase inhibitors nilotinib or sorafenib [212,236].

Generally, CPI-613 is well tolerated and has been proven to synergize with standard chemotherapeutic drugs. Advantageously, the patients treated with CPI-613 showed relatively few adverse or highly toxic side effects throughout all the published clinical studies. The main events observed include fatigue, diarrhea, vomiting, electrolyte imbalance, neutropenia/lymphopenia, and sensorial neuropathy. However, a retrospective study by Anderson et al. revealed an increased incidence of acute kidney injury [237]. The acute kidney injury, which was preferentially observed in the elderly, was reversible and of moderate severity, nonetheless. Furthermore, as assessed in a Phase I clinical trial [238], CPI-613 together with 5-FU in patients with previously treated metastatic CRC were shown to have a tolerable safety profile, but signs of kidney injury due to dysregulated creatinine levels were observed. A causal link could not be established, but it is hypothesized that the acute kidney injury was indeed related to the mode of action of CPI-613 [237]. Due to the increased levels of ROS and the shift to ammoniagenesis upon impaired glutamine flux, the highly metabolically active renal epithelium is destructively affected [237,239].

Summing up, CPI-613 is a promising building block in the treatment of a variety of cancer types. CPI-613 was shown to re-sensitize chemoresistant tumor cells in vitro and in vivo and to improve the therapeutic outcome of standard chemotherapeutic regimens, also in pre-treated patients [212,216,223,236]. By combining drugs, which target different hallmarks of cancer, the development of chemoresistance is furthermore circumvented.

## 5. Conclusions and Remarks

As pointed out above, specific metabolic alterations are found in CRC cells due to malignant transformation, which unveiled several new targets for small molecule inhibitors. Many of these compounds are in advanced clinical trials and it is foreseeable that some will be approved within the next few years. Particularly CPI-613, which is at a very advanced stage of clinical development, is a promising drug candidate for both hematological malignancies and solid tumors. To date, CPI-613 has been successfully tested as a monotherapy or in combination with established chemotherapeutics. CPI-613 may also synergistically act with pharmacological inhibitors targeting the DNA damage response proteins poly(ADP-ribose) polymerase-1 (PARP-1) and PARP-2 [240]. PARP inhibitors, such as Olaparib or Talazoparib, are already approved for the treatment of breast and ovarian cancer [241]. Interestingly, the GLS inhibitor CB-839 will be tested together with Talazoparib in a clinical Phase I/II trial, which currently recruits patients with solid tumors, including CRC (NCT03875313). Furthermore, PARP-1 is known to be overexpressed in human CRC specimens and was shown to promote CRC progression in vivo [242]. It is thus tempting to speculate that CPI-613 may also synergize with PARP inhibitors in CRC, by the dual targeting of CRC metabolism and the DNA damage response. Together with the recently approved immune checkpoint inhibitors [243], our treatment options to fight advanced and metastatic CRC are further expanded, which will help to improve the long-term survival rates of patients suffering from this fatal disease.

## Figures and Tables

**Table 1:** Selected clinical trials for solid tumors including the colorectal cancer (CRC) of small molecules that target the TCA cycle.

Drug	Phase	Condition(s)	Combination Therapy	Identification No.
CPI-613	I	Advanced or Metastatic Cancer, Metastatic Cancer, Lymphoma, Solid Tumors, Advanced Malignancies	-	NCT00741403
	I	(Advanced or Metastatic) CRC	5-FU	NCT02232152
	I/II	Cancer, Pancreatic Cancer	Gemcitabine	NCT00907166
	II	Cancer	-	NCT01832857
DCA	I	Recurrent or Metastatic Solid Tumors	-	NCT00566410
AG-221	I/II	Solid Tumor, Glioma, Angioimmunoblastic T-cell Lymphoma, Intrahepatic Cholangiocarcinoma, Chondrosarcoma	-	NCT02273739
	I/II	Refractory or Recurrent Malignancies	-	NCT02813135
CB-839	I/II	Solid Tumor, Clear Cell Renal Cell Carcinoma, (Triple-Negative) Breast Cancer, CRC, (Clear Cell) Renal Cell Carcinoma	Talazoparib	NCT03875313
	I/II	CRC, Solid Tumor	Capecitabine	NCT02861300
	I/II	(Metastatic or Refractory) CRC	Panitumumab + Irinotecan	NCT03263429
	I/II	Solid Tumors, Non-Small Cell Lung Cancer, CRC	Palbociclib	NCT03965845

## Abbreviations

2-HG	2-hydroxyglutarate
5-FU	5-fluorouracil
ACC	acetyl CoA carboxylase
2-HG	2-hydroxyglutarate
ADP	adenosine diphosphate
αKG	α ketoglutarate
AKT	protein kinase B
AML	acute myeloid leukemia
AMPK	5’ AMP-activated protein kinase
Bax	Bcl-2-associated X protein
BW	body weight
CAF	cancer-associated fibroblast
CD	cluster of differentiation
CDK	cyclin-dependent kinase
CIMP	CpG island methylator phenotype
COX	cyclooxygenase
CRC	colorectal cancer
CSC	cancer stem cell
CYP	cytochrome P450
DCA	dichloroacetate
dMMR	DNA mismatch repair system
DNA	deoxyribonucleic acid
E4BP	eIF4E binding protein
ECM	extracellular matrix
EGFR	endothelial growth factor receptor
EMA	European Medicines Agency
EMT	epithelial–mesenchymal transition
ERK	extracellular-signal regulated kinases
FAD	flavin adenine dinucleotide
FDA	Food and Drug Administration
FOLFIRI	fluorouracil, leucovorin and irinotecan
FOLFIRINOX	fluorouracil, leucovorin, irinotecan and oxaliplatin
FOLFOX	fluorouracil, leucovorin and oxaliplatin
G6PD	glucose-6-phosphate dehydrogenase
GLS	glutaminase
GLUD	glutamate dehydrogenase
GLUT	glucose transporter
GOT	glutamic-oxaloacetic transaminase
GTP	glutamate pyruvate transaminase
GTP	guanosine-5’-triphosphate
HIF	hypoxia-inducible factor
HK	hexokinase
HRE	hypoxia response element
IBD	inflammatory bowel disease
IC50	inhibitory concentration (50%)
IDH	isocitrate dehydrogenase
IGF	insulin-like growth factor
KGDH	α-ketoglutarate dehydrogenase complex
LA	α-lipoic acid
LC3B	microtubule-associated protein
LDHA	lactate dehydrogenase A
MAPK	mitogen-activated protein kinase
MCT	monocarboxylate transporter
MDR	multidrug resistant protein
MGMT	O6-methylguanine-DNA methyltransferase
MOM	mitochondrial outer membrane
MPC	mitochondrial pyruvate carrier
MSI	microsatellite instable
MSS	microsatellite stable
mtDNA	mitochondrial DNA
MTD	maximum tolerable dose
MTHFD2	methylenetetrahydrofolate dehydrogenase
mTOR	mammalian target of rapamycin
MYC	myelocytomatosis
NFκB	nuclear factor ‘kappa-light-chain-enhancer’ of activated B-cells
Nrf	nuclear factor erythroid-2-related factor
OXPHOS	oxidative phosphorylation
PARP	poly(ADP-ribose) polymerase
PDGF	platelet-derived growth factor
PGC	peroxisome proliferator-activated receptor gamma coactivator
PDC	pyruvate dehydrogenase complex
PDCD4	programmed cell death protein
PDK	pyruvate dehydrogenase kinase
PEG2	prostaglandin E2
PEG	polyethylene glycol
PET	positron emission tomography
PFK	phosphofructokinase
PHD	prolyl hydroxylase domain protein
PI3K	phosphoinositide 3-kinases
PIP2	phosphatidylinositol-4,5-bisphosphate
PIP3	phosphatidyl-inositol-3,4,5-trisphosphate
PKM	pyruvate kinase
PPP	pentose phosphate pathway
ROS	reactive oxygen species
RTK	receptor tyrosine kinases
S6K1	p70S6 Kinase 1
S9	supernatant 9000 g,—
SCO	synthesis of cytochrome c oxidase
SLC	solute carrier
SREBP	sterol responsive element binding protein
TAM	tumor associated macrophages
TCA	tricarboxylic acid cycle
T_eff_ cell	effector T cell
TGF	transforming growth factor
TME	tumor microenvironment
TIGAR	TP53-inducible glycolysis and apoptosis regulator
T_reg_ cell	regulatory T cell
VEGF	vascular endothelial growth factor
